# Immunogenic Cell Death and Metabolic Reprogramming in Cancer: Mechanisms, Synergies, and Innovative Therapeutic Strategies

**DOI:** 10.3390/biomedicines13040950

**Published:** 2025-04-12

**Authors:** Jie Jiang, Yan Yan, Chunhui Yang, Hong Cai

**Affiliations:** Department of Clinical Laboratory, The Second Hospital of Dalian Medical University, Dalian 116023, China; yigaozhong14@126.com (J.J.); y_july@163.com (Y.Y.)

**Keywords:** immunogenic cell death, metabolic reprogramming, tumor microenvironment, cancer immunotherapy

## Abstract

Immunogenic cell death (ICD) is a promising cancer therapy where dying tumor cells release damage-associated molecular patterns (DAMPs) to activate immune responses. Recent research highlights the critical role of metabolic reprogramming in tumor cells, including the Warburg effect, oxidative stress, and lipid metabolism, in modulating ICD and shaping the immune microenvironment. These metabolic changes enhance immune activation, making tumors more susceptible to immune surveillance. This review explores the molecular mechanisms linking ICD and metabolism, including mitochondrial oxidative stress, endoplasmic reticulum (ER) stress, and ferroptosis. It also discusses innovative therapeutic strategies, such as personalized combination therapies, metabolic inhibitors, and targeted delivery systems, to improve ICD efficacy. The future of cancer immunotherapy lies in integrating metabolic reprogramming and immune activation to overcome tumor immune evasion, with multi-omics approaches and microbiome modulation offering new avenues for enhanced treatment outcomes.

## 1. Introduction

**Figure 1:**
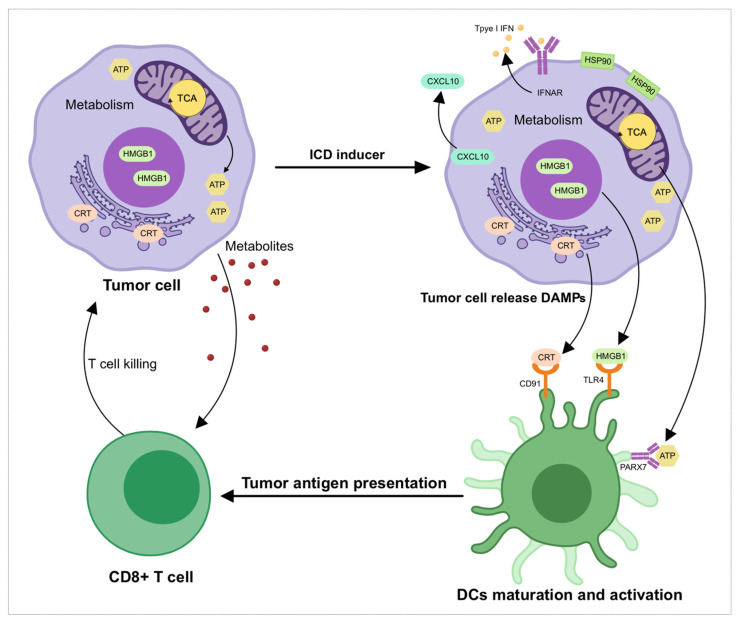
Molecular mechanisms of metabolic reprogramming and immune activation in immunogenic cell death (ICD). Cancer cells undergoing ICD release critical signals, including calreticulin (CRT, an “eat me” signal), high-mobility group box 1 (HMGB1), other damage-associated molecular patterns (DAMPs), and metabolites. Released DAMPs (HMGB1, ATP, CRT) recruit and activate dendritic cells (DCs), which promote antigen uptake and mature, presenting these tumor-associated antigens (TAAs) to CD8+ T-cell, resulting in tumor-specific immune activation. Metabolites released by tumor cells modulate dendritic cell activation and T-cell infiltration and functionality. Metabolic reprogramming influences ICD by modulating DAMPs’ release, metabolite secretion, and shaping immune cell function in the tumor microenvironment (TME), including DC maturation and T-cell activities, thus affecting overall tumor immunogenicity. Abbreviations: DAMPs, damage-associated molecular patterns; CRT, calreticulin; DCs, dendritic cells; HMGB1, high-mobility group box 1; ICD, immunogenic cell death; TAAs, tumor-associated antigens; TME, tumor microenvironment; TCA, tricarboxylic acid cycle, ATP, adenosine triphosphate; CXCL10, C-X-C motif chemokine ligand 10; PARX7, Parkinson’s disease protein 7; TLR4, toll-like receptor 4; CD91, low-density lipoprotein receptor-related protein 1; IFN, interferon; IFNAR, interferon alpha receptor; HSP90, heat shock protein 90.

Immunogenic cell death (ICD) has emerged as a critical and complex mechanism in the context of cancer immunotherapy [1]. Traditionally, cell death was classified into apoptosis, a process considered immunologically silent, and necrosis, which was thought to provoke an immune response [2]. However, recent discoveries have highlighted that apoptotic cell death, under certain conditions, can indeed stimulate a robust immune response, thus challenging the conventional understanding [3]. This phenomenon, known as ICD, has profound implications for cancer treatment [4]. Unlike typical apoptosis, which generally evades immune surveillance, ICD leads to the release of several damage-associated molecular patterns (DAMPs) from dying cells [5]. These DAMPs, including high-mobility group box 1 (HMGB1) [6], calreticulin (CRT) [7], and other antigens, serve as critical signals to recruit and activate dendritic cells (DCs) [8]. Immature DCs promote the uptake of these antigens and mature, subsequently presenting them to cytotoxic T lymphocytes (CTLs), thus promoting tumor-specific immune responses. Additionally, metabolites released from cancer cells modulate the immune environment, influencing T-cell function and responsiveness to immune checkpoint blockade therapies, such as programmed death-1(PD-1) and programmed cell death ligand 1(PD-L1) inhibitors (Figure 1).

ICD is intricately linked with metabolic reprogramming, which has become a hallmark of cancer progression [9]. Cancer cells, due to their altered metabolic pathways, often rely on glycolysis for adenosine triphosphate (ATP) production even in the presence of sufficient oxygen, a phenomenon referred to as the Warburg effect [10]. This shift toward glycolysis enables rapid cell growth and survival under nutrient-deprived and hypoxic conditions often present within the tumor microenvironment (TME) [11,12]. While metabolic reprogramming supports cancer cell proliferation and survival, it also profoundly impacts the tumor immune microenvironment [13]. The metabolic changes in tumor cells, such as increased glycolytic activity and mitochondrial dysfunction, not only contribute to the malignant phenotype but also influence the behavior of immune cells in the tumor niche [14].

Recent research has illuminated the intricate connection between ICD and metabolic alterations in tumor cells [15]. Metabolic reprogramming in cancer can influence the generation and release of DAMPs, thus modulating the immune response [16]. For example, oxidative stress [17] and ER stress [18], commonly observed in tumor cells undergoing ICD, can trigger the release of immune-stimulating molecules, including CRT and ATP [19]. These molecules act in concert to activate DCs and other immune cells, leading to the priming of tumor-specific CTLs [20]. Furthermore, changes in cellular metabolism, such as increased lactate production due to glycolysis, can create an immunosuppressive microenvironment, limiting the effectiveness of ICD and immune responses [21].

Given the complex interplay between ICD and metabolic reprogramming, there is a growing interest in exploring innovative therapeutic strategies that target both metabolic pathways and immune activation [22]. Such approaches have the potential to enhance the efficacy of cancer immunotherapies by improving the immunogenicity of cell death while overcoming the immunosuppressive TME [23]. Combination therapies that integrate ICD inducers with metabolic inhibitors, immune checkpoint blockade, or nanomedicine-based strategies offer exciting opportunities to enhance tumor-specific immune responses and improve patient outcomes [24].

Although numerous reviews have addressed ICD and metabolic reprogramming independently, our review uniquely integrated recent insights into multiple ICD modalities, including apoptosis, ferroptosis, pyroptosis, and necroptosis, with comprehensive metabolic alterations and clinical translational perspectives. Furthermore, we discussed advanced multi-omics clinical applications and emerging genetic editing strategies (such as Clustered regularly interspaced short palindromic repeats—CRISPR-associated protein 9, CRISPR/Cas9) targeting cancer metabolism to enhance ICD. These integrative and translational insights significantly distinguish our review from previously published articles, providing readers with a cutting-edge, comprehensive synthesis of this rapidly evolving field.

## 2. Molecular Mechanisms and Features of ICD

ICD encompasses various forms of regulated cell death beyond immunogenic apoptosis, notably ferroptosis, pyroptosis, and necroptosis, each characterized by distinct cellular stress signals and immunogenic consequences [25]. Ferroptosis is an iron-dependent form of regulated cell death triggered by lipid peroxidation, intimately linked to lipid metabolism pathways. Ferroptosis-induced lipid peroxides serve as potent DAMPs, enhancing DC maturation and T-cell activation [26]. Pyroptosis, induced through inflammasome activation, leads to gasdermin-mediated plasma membrane rupture and release of pro-inflammatory cytokines like interleukin-1β (IL-1β), interleukin-18 (IL-18) and other DAMPs, facilitating potent immune responses. This pathway is closely associated with metabolic stress, especially mitochondrial reactive oxygen species (ROS) generation [27]. Necroptosis, mediated by receptor-interacting protein kinase 1/receptor-interacting protein kinase 3/mixed lineage kinase domain-like protein (RIPK1/RIPK3/MLKL) signaling cascade, is often initiated by metabolic perturbations, such as ATP depletion, oxidative stress, or glycolysis inhibition, resulting in rapid release of inflammatory DAMPs [28]. Each of these cell death modes demonstrates a unique but overlapping link with metabolic reprogramming, where alterations in energy metabolism, ROS production, and lipid metabolism distinctly modulate immunogenic outcomes.

### 2.1. Mechanisms of ICD Activation

ICD is initiated by various cellular stressors, including chemotherapy-induced DNA damage, ER stress, ROS production, mitochondrial dysfunction, and metabolic perturbations [29]. These cellular insults trigger downstream signaling cascades that culminate in regulated forms of cell death, subsequently leading to characteristic hallmarks of ICD, such as the exposure of CRT on the cell surface and the active release of DAMPs like HMGB1 and ATP, essential for the immune activation cascade, which play a pivotal role in activating the immune system [30]. One of the first key events in ICD is the translocation of CRT from the ER to the surface of dying cells. This relocation of CRT acts as an “eat me” signal, prompting the phagocytosis of dying cells by DCs and macrophages [31]. Once internalized, tumor antigens are processed and presented to T-cells, triggering adaptive immune responses [32]. In parallel, ATP, a crucial energy molecule, is released from dying cells. Extracellular ATP serves as a “find me” signal that recruits immune cells, particularly DCs, to the site of cell death [33]. ATP binding to P2X Purinoceptor 7 (P2X7) receptors on DCs activates the NLR family pyrin domain-containing protein 3 (NLRP3) inflammasome, promoting the release of pro-inflammatory cytokines like IL-1β, which further aids in the maturation of DCs and enhances their antigen-presenting capabilities [34]. Additionally, HMGB1, a nuclear protein, is released from the nucleus during cell death, where it binds to receptors such as toll-like receptor 4 (TLR4) and receptor of advanced glycation endproduct (RAGE) on DCs, promoting pro-inflammatory cytokine production and facilitating T-cell activation [35]. These DAMPs, including CRT, ATP, and HMGB1, collectively drive a robust immune response, ensuring that the dying tumor cells are recognized and attacked by the immune system. In this way, ICD not only eliminates tumor cells but also induces a potent immune response, promoting the development of tumor-specific immunity.

### 2.2. Core Features of ICD

The core features of ICD are defined by a unique combination of signals that ensure the death of tumor cells is immunologically active, initiating a potent immune response [36]. One of the defining characteristics of ICD is the “immunogenic triad”, which includes the surface exposure of CRT, the release of extracellular ATP, and the secretion of HMGB1 [37]. These three signals act synergistically to promote immune activation and ensure that the immune system recognizes and responds to the tumor. The exposure of CRT on the surface of tumor cells provides a signal for immune cells to phagocytose the dying cells, while ATP and HMGB1 further amplify the immune response, enhancing dendritic cell maturation and the activation of CTLs [38]. Moreover, the diversity of tumor antigens released during ICD, including tumor-associated antigens (TAAs), neoantigens, and viral antigens, is essential in overcoming tumor heterogeneity and immune evasion [39,40]. This antigenic diversity enables the immune system to generate a broader immune response, enhancing the efficacy of immunotherapies, particularly in terms of CD8⁺ CTL activation [41]. Another critical aspect of ICD is its ability to synergize with other therapeutic approaches, such as chemotherapy and radiotherapy [42]. These therapies induce stress in tumor cells, which can lead to ICD and enhance the release of DAMPs, thus boosting the overall immune response [43]. However, negative feedback mechanisms, such as the activation of the mevalonate pathway leading to increased cholesterol synthesis, can dampen the immunogenic effects of ICD. Understanding and overcoming such regulatory mechanisms is key to maximizing the therapeutic potential of ICD.

## 3. Metabolic Regulation of ICD

**Figure 2:**
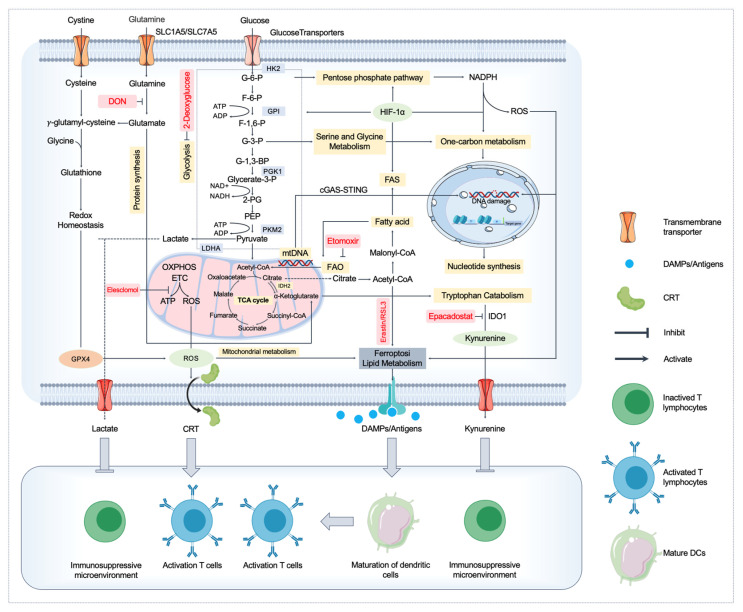
Metabolic pathways regulating immune responses in immunogenic cell death (ICD) and targeting strategies. Cancer cell metabolism, including glycolysis, glutamine metabolism, lipid metabolism, ferroptosis, and mitochondrial oxidative phosphorylation (OXPHOS), regulate immune responses by modulating the release of reactive oxygen species (ROS), mitochondrial DNA (mtDNA), lipid peroxidation products, and other critical damage-associated molecular patterns (DAMPs). Specifically, ROS generated from mitochondrial dysfunction and ferroptosis facilitate the externalization and release of DAMPs, thus activating dendritic cells (DCs) and enhancing T-cell priming and infiltration. Ferroptosis and lipid metabolism-derived lipid peroxides directly act as potent immunostimulants. Current and promising therapeutic strategies targeting these metabolic pathways include glycolysis inhibitors (e.g., 2-deoxyglucose), glutaminolysis inhibitors (e.g., DON), ferroptosis inducers (e.g., erastin, RSL3), and indoleamine 2,3-dioxygenase 1 (IDO1) inhibitors (e.g., epacadostat), aiming to enhance ICD-mediated antitumor immunity and overcome immune resistance in cancer therapy. Abbreviations: ICD, immunogenic cell death; DAMPs, damage-associated molecular patterns; ATP, adenosine triphosphate; ADP, adenosine diphosphate; G-6-P, glucose-6-phosphate; F-6-P, fructose-6-phosphate; F-1,6-P, fructose-1,6-bisphosphate; G-3-P, glyceraldehyde-3-phosphate; 1,3-BP, 1,3-bisphosphoglycerate; 3-PG, 3-phosphoglycerate; 2-PG, 2-phosphoglycerate; PEP, phosphoenolpyruvate; HK2, hexokinase 2; GPI, glucose-6-phosphate isomerase; PGK1, phosphoglycerate kinase 1; PKM2, pyruvate kinase M2; LDHA, lactate dehydrogenase A; OXPHOS, oxidative phosphorylation; ETC, electron transport chain; ROS, reactive oxygen species; GSH, glutathione; mtDNA, mitochondrial DNA; cGAS-STING, cyclic GMP-AMP synthase-stimulator of interferon genes; FAS, fatty acid synthesis; FAO, fatty acid oxidation; HIF-1α, hypoxia-inducible factor-1 alpha; IDO1, indoleamine 2,3-dioxygenase 1; SLC1A5, solute carrier family 1 member 5; SLC7A5, solute carrier family 7 member 5; DON, 6-diazo-5-oxo-l-norleucine.

Metabolic reprogramming plays a crucial role in the induction and regulation of ICD. Tumor cells undergo metabolic alterations, such as increased glycolysis, oxidative stress, and lipid metabolism, which influence their ability to undergo ICD and modulate immune responses. For example, mitochondrial dysfunction and ROS accumulation in stressed cells facilitate the release of DAMPs, enhancing immune activation. Additionally, metabolic pathways like serine metabolism and lipid peroxidation can modulate the immunogenicity of cell death, promoting the recruitment of immune cells and the activation of antitumor immune responses. Targeting these metabolic pathways provides a promising strategy to enhance ICD and improve the efficacy of cancer immunotherapy (Figure 2).

### 3.1. Disruption of Mitochondrial Function and ICD

Mitochondria are essential organelles involved in energy production, cellular metabolism, and the regulation of cell death. Recent studies have revealed that mitochondrial dysfunction plays a critical role in the induction of ICD, a form of cell death that not only eliminates tumor cells but also activates immune responses [44]. Mitochondrial disruption leads to a cascade of molecular events that can trigger ICD, facilitating the release of DAMPs that engage the immune system and enhance antitumor immunity.

#### 3.1.1. Mitochondrial Dysfunction and ICD Induction

Mitochondria play a central role in maintaining cellular homeostasis by regulating oxidative phosphorylation (OXPHOS), energy production, and redox balance [45]. When mitochondrial function is disrupted, through stress conditions like oxidative damage or metabolic reprogramming, a series of events unfold that promote cell death. In the context of ICD, mitochondrial dysfunction leads to the release of various DAMPs, such as mitochondrial DNA (mtDNA), ATP, and proteins like cytochrome c, that serve as potent immune activators [46]. Mitochondrial damage can trigger the intrinsic apoptotic pathway by promoting the release of pro-apoptotic factors like cytochrome c and Smac/DIABLO into the cytoplasm [47]. However, under conditions of stress or in the case of certain chemotherapies, this mitochondrial dysfunction can also lead to the release of immunogenic signals. The presence of extracellular ATP can recruit DCs and other immune cells to the site of cell death, initiating immune priming. Furthermore, the release of mitochondrial proteins like HMGB1 can further enhance the immune response by interacting with toll-like receptors (TLRs) on DCs, promoting the maturation of these cells and stimulating T-cell activation [48].

#### 3.1.2. Mitochondrial ROS and ICD

Mitochondria are also major sources of ROS, which are crucial for maintaining redox balance in the cell. When mitochondrial function is disrupted, ROS levels can increase dramatically, leading to oxidative stress [49]. This ROS accumulation can damage cellular components, including lipids, proteins, and DNA, as well as contribute to the initiation of cell death. In the case of ICD, mitochondrial ROS not only induce cell death directly but also enhance the immunogenicity of the dying cells [50]. ROS accumulation in stressed or dying cells plays a pivotal role in ICD by facilitating the exposure of DAMPs on the cell surface. For example, ROS are involved in the translocation of CRT from the ER to the cell surface, where it acts as an “eat me” signal for phagocytosis by DCs [51]. Moreover, ROS can activate the NLRP3 inflammasome, leading to the production of pro-inflammatory cytokines like IL-1β. These cytokines further stimulate immune cells, such as DCs and T-cells, enhancing the antitumor immune response [52].

Furthermore, ROS can activate the mitochondrial apoptotic pathway by impairing mitochondrial membrane integrity. This impairment triggers the release of mitochondrial contents into the cytoplasm, where they act as immune stimulators [53]. For example, mtDNA, when released into the cytosol, can activate the cyclic GMP-AMP synthase (cGAS)-stimulator of interferon genes (STING) pathway, which plays a central role in the induction of type I interferons and the subsequent activation of immune responses [54]. These events are pivotal in ICD as they not only promote cell death but also stimulate immune cells to recognize and attack the tumor.

#### 3.1.3. mtDNA and the cGAS-STING Pathway in ICD

The release of mtDNA during mitochondrial dysfunction is a key feature of ICD [55]. Under stress conditions, damaged mitochondria can release mtDNA into the cytoplasm, where it is recognized by the cGAS-STING pathway, a critical pathway for the induction of innate immune responses [56]. This pathway, when activated by mtDNA, triggers the production of type I interferons and other pro-inflammatory cytokines that enhance the immune response against tumor cells. Activation of the cGAS-STING pathway following the release of mtDNA serves as a potent DAMP, helping to bridge the gap between tumor cell death and immune system activation. This pathway enhances the antigen-presenting capacity of DCs, promotes the infiltration of CTLs, and strengthens the overall antitumor immune response [57]. Moreover, the activation of STING in tumor cells has been shown to increase their sensitivity to immune checkpoint blockade therapies, further underscoring the therapeutic potential of combining ICD inducers with STING-activating agents [58].

In addition to mtDNA, other mitochondrial-derived factors, such as mitochondrial peptides and proteins, can also play a role in immune activation. These factors, once released during mitochondrial dysfunction, interact with pattern recognition receptors (PRRs) on immune cells to trigger inflammatory responses that enhance the effectiveness of ICD [59]. By targeting the release and recognition of mitochondrial DAMPs, it is possible to potentiate the immunogenic effects of ICD and improve the efficacy of cancer immunotherapies.

### 3.2. Lipid Metabolism, Ferroptosis, and ICD

Lipid metabolism and ferroptosis are critical processes involved in the regulation of ICD, influencing both tumor cell survival and the immune response [29]. These pathways are closely intertwined with tumor progression and immune system modulation, with significant implications for enhancing or inhibiting the effectiveness of ICD-based cancer therapies. Below, we discuss the role of lipid metabolism and ferroptosis in ICD, providing a detailed explanation of how these processes contribute to tumor immunogenicity and immune activation.

#### 3.2.1. Lipid Metabolism and Its Role in ICD

Lipid metabolism plays a fundamental role in maintaining cellular integrity, signaling, and energy storage in both tumor and immune cells [60]. Tumor cells, due to their rapid proliferation, undergo metabolic reprogramming, which includes alterations in lipid biosynthesis [61]. This reprogramming is crucial for providing the necessary lipids for membrane biogenesis and for fueling the elevated energy demands of the tumor. Dysregulated lipid metabolism, particularly the accumulation of lipid species like phospholipids and cholesterol, is associated with cancer progression and immune suppression [62]. Alterations in lipid metabolism not only impact tumor cells directly but also shape the TME, influencing immune responses and ICD [63].

In the context of ICD, lipid metabolism has an essential role in the induction of cell death and the modulation of immune responses. For instance, tumor cells undergoing ICD can release various lipid-based DAMPs such as oxidized phospholipids and ceramide [64]. These lipid metabolites act as immunological signals that can stimulate the maturation of DCs and enhance the recruitment of immune cells like CTLs to the tumor site [65,66]. Furthermore, lipid accumulation in the TME can enhance the secretion of pro-inflammatory cytokines, promoting the activation of the immune system [67]. Studies suggest that targeting lipid metabolism pathways, such as inhibition of fatty acid synthase (FASN) or acyl-CoA synthetase long-chain family member 4 (ACSL4), could promote ICD by increasing lipid peroxidation and enhancing the immunogenicity of tumor cells [68]. Therefore, modulating lipid metabolism provides an innovative approach to boost ICD-induced tumor immunogenicity and improve the effectiveness of cancer immunotherapy.

#### 3.2.2. Ferroptosis and Its Interplay with ICD

Ferroptosis, a form of regulated cell death characterized by iron-dependent lipid peroxidation, has recently gained attention for its potential to synergize with ICD and enhance immune responses in cancer [29]. Ferroptosis is distinct from other forms of cell death, such as apoptosis and necrosis, as it involves the accumulation of lipid peroxides to toxic levels within the cell, leading to membrane rupture and cell death. This process is driven by iron metabolism, ROS generation, and the inhibition of key antioxidant defense mechanisms, particularly glutathione peroxidase 4 (GPX4) [69].

The relationship between ferroptosis and ICD is multifaceted. Both processes can lead to the release of similar DAMPs, including ATP, HMGB1, and oxidized phospholipids, that activate DCs and prime CTLs to mount an effective immune response [26]. Ferroptosis-induced cell death, through lipid peroxidation, can directly trigger the release of these immunogenic signals, which enhances immune activation and promotes a more robust antitumor response. For example, lipid peroxidation products released from dying tumor cells during ferroptosis can serve as potent signals for DCs, which facilitate the presentation of tumor antigens to T-cells, thereby enhancing T-cell priming and activation [70]. Moreover, recent studies have shown that ferroptosis can synergize with ICD by enhancing the immunogenicity of tumor cells [71]. One mechanism is the induction of lipid peroxidation through ferroptosis inducers like erastin or RSL-3. These inducers can boost the release of DAMPs, amplifying the immune response [72]. Additionally, combining ferroptosis inducers with ICD-inducing therapies, such as chemotherapy or photodynamic therapy (PDT), has been shown to significantly enhance the antitumor immune response and reduce tumor growth [73]. This dual approach exploits the immunogenic potential of both ferroptosis and ICD, making tumor cells more susceptible to immune attack.

#### 3.2.3. Lipid Peroxidation and Immune Activation

Lipid peroxidation, the process by which polyunsaturated fatty acids in cell membranes undergo oxidative degradation, is central to both ferroptosis and ICD [74]. During ferroptosis, excessive lipid peroxidation leads to the generation of toxic lipid aldehydes, such as 4-hydroxy-2-nonenal (4-HNE), which disrupt cellular membranes and activate signaling pathways that lead to cell death [75]. These lipid peroxidation products, along with oxidized phospholipids, act as potent DAMPs that activate the immune system. The release of these metabolites into the extracellular space can recruit immune cells, including DCs and macrophages, to the site of cell death. This, in turn, promotes the maturation of DCs and the activation of tumor-specific T-cells, enhancing the immunogenicity of tumor cells undergoing ICD or ferroptosis. The accumulation of lipid peroxides also plays a significant role in shaping the TME by modulating the activity of immune cells. Lipid peroxidation has been shown to influence the polarization of macrophages, promoting a pro-inflammatory phenotype that is conducive to immune activation. Moreover, ferroptosis-induced lipid peroxidation can overcome the immunosuppressive effects of the TME, which often limits the effectiveness of traditional therapies. Therefore, enhancing lipid peroxidation through ferroptosis inducers or lipid metabolism modulation represents a promising strategy to augment ICD and improve antitumor immunity.

## 4. Amino Acid Metabolism and ICD

Amino acid metabolism plays a pivotal role in the regulation of cancer cell behavior and immune responses, significantly influencing the induction and progression of ICD [76]. Tumor cells exhibit altered amino acid metabolism to support rapid growth and survival under stressful conditions. This metabolic reprogramming not only facilitates tumor progression but also impacts the immune landscape within the TME, which can enhance or inhibit the efficacy of ICD-based therapies [77]. Here, we discuss the key aspects of amino acid metabolism and its involvement in ICD, supported by recent studies.

### 4.1. Glutamine Metabolism

Glutamine is a vital amino acid in cancer metabolism, often referred to as the “fuel of cancer” due to its role in sustaining various metabolic processes such as nucleotide synthesis, redox balance, and energy production [78]. Tumor cells rely heavily on glutamine for these functions, making it a key player in cellular metabolism and immune modulation [79]. Glutamine deprivation can induce metabolic stress and influence the immune response. Studies have shown that glutamine deprivation can activate the unfolded protein response (UPR), which in turn triggers ICD by releasing DAMPs such as CRT and HMGB1 [80]. Furthermore, glutamine metabolism plays a role in immune suppression within the TME. High levels of glutamine in the tumor can promote the accumulation of regulatory T-cells (Tregs) and suppress effector T-cells [81], which hampers the effectiveness of ICD. Targeting glutamine metabolism with inhibitors such as 6-diazo-5-oxo-L-norleucine (DON) has shown promise in sensitizing tumors to ICD by disrupting the glutamine-driven immune suppression and enhancing tumor immunogenicity [82]. Thus, modulating glutamine metabolism could potentially enhance ICD and improve the efficacy of immunotherapy by reducing immune tolerance and promoting immune activation.

### 4.2. Tryptophan Catabolism and Immune Suppression

Tryptophan metabolism is another critical pathway in the immune regulation of cancer [83]. The enzyme indoleamine 2,3-dioxygenase 1 (IDO1) catalyzes the catabolism of tryptophan into immunosuppressive metabolites such as kynurenine. IDO1 expression is commonly upregulated in various cancers, and it plays a key role in creating an immunosuppressive microenvironment [84]. The depletion of tryptophan in the TME inhibits T-cell proliferation and promotes the differentiation of Tregs, which dampens antitumor immune responses [85]. Moreover, the accumulation of kynurenine and its downstream metabolites can directly impair the function of DCs, further hindering immune activation [86]. Recent studies have demonstrated that the inhibition of IDO1, either through small molecules like epacadostat or monoclonal antibodies, can restore T-cell activity and enhance the immune response in tumors [87]. By combining IDO1 inhibition with ICD inducers, it is possible to not only restore immune function but also amplify the antitumor immune response, overcoming immune suppression and enhancing the overall efficacy of cancer immunotherapy. This dual approach could significantly improve the outcomes of ICD-based treatments by overcoming the metabolic blockade imposed by tryptophan catabolism [88].

### 4.3. Serine and Glycine Metabolism

Serine and glycine metabolism plays a pivotal role in one-carbon metabolism, which is essential for DNA and nucleotide biosynthesis [89]. Tumor cells have elevated requirements for serine and glycine to support rapid cell division and proliferation. This metabolic alteration contributes to tumor growth but also affects the immune response within the TME [90]. Serine and glycine are used in the production of S-adenosylmethionine (SAMe), which is involved in methylation reactions, and thus impact epigenetic regulation [91]. In the context of ICD, studies have shown that alterations in serine and glycine metabolism can influence immune cell activation. Serine depletion has been found to increase tumor cell vulnerability to immune-mediated killing by enhancing oxidative stress [92]. Serine metabolism plays a critical role in regulating effector T-cell responses by providing glycine and one-carbon units for nucleotide biosynthesis, which supports T-cell proliferation. Restricting serine impairs T-cell expansion during immune challenges without affecting overall immune cell homeostasis, highlighting serine as a key immunometabolite that modulates adaptive immunity [93]. Additionally, targeting the serine biosynthetic pathway, using inhibitors like PH755, may improve the immune response by promoting tumor cell death and enhancing the release of DAMPs such as CRT and ATP [94]. Depriving cancer cells of serine leads to mitochondrial dysfunction, causing the accumulation of mtDNA in the cytosol. This triggers the activation of the cGAS-STING signaling pathway, which stimulates immune responses targeting the tumor. These immune responses can be further enhanced by PD-1-targeted therapy [95]. By manipulating serine and glycine metabolism, it is possible to not only induce stress in tumor cells but also enhance the immunogenicity of ICD, which could lead to improved tumor recognition and enhanced immune activation.

## 5. Immune Microenvironment Regulation by Metabolites

The TME is profoundly influenced by the metabolic state of both tumor cells and immune cells. One of the key metabolic factors in this dynamic is extracellular ATP [96]. Released from dying tumor cells during ICD, ATP serves as both a damage-associated molecular pattern (DAMP) and a chemotactic signal for immune cells. ATP promotes the maturation and activation of DCs through the P2X7 receptor, enhancing antigen presentation and facilitating T-cell priming [97]. Furthermore, ATP activates the NLRP3 inflammasome, which drives the secretion of pro-inflammatory cytokines like IL-1β, further amplifying immune activation [98]. However, the metabolic state of the TME can also lead to immunosuppressive effects, particularly through the accumulation of lactate [99]. As tumor cells rely on glycolysis for energy production, excess lactate is produced, acidifying the microenvironment [100]. This lactate buildup can inhibit T-cell function and promote the polarization of macrophages into the immunosuppressive M2 phenotype [101]. By targeting lactate metabolism or modulating lactate efflux, such as through inhibition of the MCT1 transporter, it is possible to reduce the immunosuppressive effects of lactate and enhance the efficacy of ICD [102]. Another critical metabolic pathway involved in immune regulation is tryptophan metabolism, particularly the enzyme IDO1, which accelerates tryptophan catabolism in tumor cells. This metabolic alteration depletes tryptophan in the microenvironment, suppressing T-cell proliferation and promoting immune evasion [90]. ICD can counteract this immune suppression by inhibiting IDO1 activity, thereby restoring T-cell function and improving immune activation [103]. Targeting these metabolic pathways provides a promising approach to modulate the immune microenvironment, enhancing the efficacy of ICD and improving the outcomes of cancer immunotherapies.

## 6. Targeting Metabolism to Enhance ICD and Cancer Immunotherapy

The interplay between metabolism and ICD offers significant therapeutic potential for enhancing cancer immunotherapy. Tumor cells undergo extensive metabolic reprogramming, which not only supports their rapid growth but also alters the TME, influencing immune cell function. Recent research has highlighted the importance of various metabolic pathways, including serine metabolism, in regulating immune responses and the effectiveness of ICD. By targeting these metabolic pathways, we can enhance the immunogenicity of cell death, activate immune responses, and improve the efficacy of cancer immunotherapies. Below, we explore how manipulating metabolic pathways can synergize with ICD to improve tumor treatment outcomes.

### 6.1. Metabolic Reprogramming to Enhance ICD Immunogenicity

Metabolic reprogramming plays a crucial role in enhancing the immunogenicity of cell death, especially in the context of ICD. One of the most powerful mechanisms is the induction of mitochondrial oxidative stress or ferroptosis. Both processes increase the production of ROS in tumor cells, which can disrupt mitochondrial function and induce cellular damage. Elevated ROS levels not only impair tumor cell viability but also promote the release of key DAMPs such as ATP, HMGB1, and CRT. These DAMPs are vital in stimulating the immune system by activating DCs and enhancing T-cell infiltration into the tumor site, thereby increasing the efficiency of antitumor immune responses. Ferroptosis, a form of programmed cell death driven by lipid peroxidation, also synergizes with ICD by triggering the release of lipid peroxidation products that act as immune stimulants. This combination of oxidative stress and ferroptosis amplifies the immunogenicity of tumor cells, making them more susceptible to immune surveillance and improving the overall efficacy of ICD-based therapies [104].

Another promising approach to enhancing ICD immunogenicity involves targeting cholesterol metabolism in tumor cells [105]. Cholesterol is crucial for maintaining membrane integrity and facilitating cellular signaling, and its metabolism is often altered in cancer cells. Targeting cholesterol metabolism may improve the effectiveness of ICD by enhancing the release of DAMPs such as ATP and CRT, which in turn boosts immune cell activation. In addition, cholesterol-lowering strategies can enhance the efficacy of immunotherapies like PDT [106]. PDT relies on the generation of ROS to damage tumor cells, and the modulation of cholesterol metabolism can increase the vulnerability of tumor cells to PDT by promoting the release of immune-activating signals [107]. By modulating tumor metabolism, especially through oxidative stress and cholesterol regulation, the immunogenic potential of cell death can be significantly enhanced, thereby improving the therapeutic outcomes of ICD-based cancer therapies.

### 6.2. Targeted Delivery Systems for ICD and Metabolic Regulation

One of the most promising strategies to enhance the efficacy of ICD is the development of targeted delivery systems that can precisely deliver ICD inducers and metabolic modulators to the tumor site [108]. Traditional therapeutic agents often struggle with selective targeting, leading to systemic toxicity and suboptimal therapeutic outcomes. However, smart nanocarriers have emerged as an effective tool to overcome these limitations. These nanocarriers can be engineered to respond to specific metabolic features of the TME, such as changes in pH, ROS, or hypoxia, ensuring that the delivery of therapeutic agents is both efficient and tumor-specific [109]. For instance, pH-sensitive nanoparticles can release ICD inducers or metabolic regulators in the acidic TME, thereby maximizing therapeutic effects while minimizing damage to healthy tissues [110]. Additionally, ROS-responsive systems can trigger the release of these agents when oxidative stress is elevated, which is a common feature of many cancer cells undergoing ICD [111]. By targeting the unique metabolic and stress characteristics of the TME, these advanced delivery systems enhance the release of DAMPs and improve immune responses, leading to more effective tumor eradication and reduced risk of relapse.

These targeted delivery systems also have the potential to simultaneously deliver multiple agents, such as chemotherapy drugs, immune checkpoint inhibitors, or metabolic modulators, to achieve synergistic therapeutic effects [112]. For example, the combination of ICD inducers with agents that inhibit tumor-specific metabolic pathways, like glycolysis or fatty acid synthesis, can enhance tumor cell death while also boosting the immune response. By delivering these therapeutic agents in a controlled and targeted manner, smart nanocarriers can increase the specificity and potency of ICD-based therapies, improving the overall outcome of cancer treatment [113]. The precise delivery of metabolic modulators further ensures that the immune microenvironment is optimally primed for ICD, leading to enhanced antitumor immunity and potentially overcoming the immunosuppressive TME that often limits the effectiveness of traditional therapies [114].

### 6.3. Combination Therapies: Synergizing ICD with Metabolic Inhibitors

Combining ICD inducers with metabolic inhibitors represents a highly promising strategy to enhance the effectiveness of cancer immunotherapy [115]. Tumor metabolism plays a crucial role in modulating the immune response within the TME. For instance, tumors often rely heavily on glycolysis, even in the presence of oxygen, a phenomenon known as the Warburg effect. This metabolic adaptation not only supports rapid tumor growth but also contributes to an immunosuppressive environment by increasing the accumulation of metabolites like lactate, which inhibit T-cell function and promote immune evasion [116]. By combining glycolysis inhibitors, such as 2-deoxyglucose or oxamate, with ICD inducers, the lactate accumulation in the TME can be reduced, thus alleviating the immunosuppressive effects and promoting a more favorable environment for immune cell infiltration and activation [117]. This combination approach increases the efficacy of ICD by enhancing the immune response while directly targeting tumor metabolism.

Another promising combination strategy involves the use of immune checkpoint inhibitors in conjunction with ICD inducers. Immune checkpoint molecules, such as PD-1 and PD-L1, are often upregulated in the TME and act to suppress T-cell activity, contributing to immune evasion. By using inhibitors of these checkpoint molecules, such as pembrolizumab or nivolumab, alongside ICD inducers, the immune system’s ability to recognize and eliminate tumor cells is greatly enhanced [9]. Furthermore, combining ICD with IDO1 inhibitors can counteract the immunosuppressive effects of tryptophan catabolism in tumors, which inhibits T-cell activity and promotes immune tolerance [118]. IDO1 inhibitors, such as epacadostat, can restore T-cell function and further amplify the immune response initiated by ICD [119]. The synergistic combination of ICD inducers with metabolic inhibitors or immune checkpoint blockers holds great potential for overcoming the immunosuppressive TME and improving the therapeutic outcomes of cancer immunotherapy.

### 6.4. Personalized Treatment Approaches

Personalized treatment approaches that integrate metabolic profiling and ICD induction hold significant promise in optimizing cancer therapies. Tumors exhibit considerable metabolic heterogeneity, with different subtypes displaying distinct metabolic characteristics such as increased glycolysis or glutamine dependence. By profiling the metabolic state of a patient’s tumor, it becomes possible to select the most effective ICD-inducing therapies tailored to the tumor’s specific metabolic profile [120]. For example, in tumors characterized by hyperglycolysis, combining ICD inducers with adenosine 5′-monophosphate-activated protein kinase (AMPK) activators or glycolytic inhibitors, such as metformin, may enhance immune responses by inhibiting the Warburg effect and promoting tumor cell death [121]. AMPK activators also induce autophagy, which can promote the release of DAMPs and further enhance immune activation. Personalized treatment approaches also extend to the development of vaccines based on ICD-induced tumor antigens [120]. These vaccines, which are derived from tumor antigens released during the ICD process, can be combined with metabolic regulators to promote long-term immune memory. By customizing immunotherapies based on both the tumor’s metabolic characteristics and the immune response to ICD, personalized approaches offer the potential for more effective, targeted treatments that improve patient outcomes [122].

Additionally, personalized treatment strategies can be optimized by using multi-omics technologies, including genomics, transcriptomics, proteomics, and metabolomics, to comprehensively analyze the metabolic and immune profiles of tumors. This integration of multi-omics data allows for a deeper understanding of the tumor’s metabolic reprogramming and immune landscape, facilitating the selection of the most suitable ICD-based therapies [123]. Furthermore, molecular imaging techniques can be employed to monitor real-time metabolic changes and assess the tumor’s response to ICD-based therapies. This dynamic monitoring enables adjustments to treatment regimens based on the tumor’s evolving metabolic profile, ensuring that therapeutic interventions remain effective throughout the course of treatment [124]. The integration of personalized vaccines, metabolic profiling, and ICD-based therapies represents a transformative approach to cancer treatment, enabling the development of more precise and effective therapies tailored to the unique characteristics of each patient’s tumor.

### 6.5. Omics Approaches for Metabolic Reprogramming in Clinical Application

The development of “omics” technologies, including genomics, transcriptomics, proteomics, and metabolomics, has greatly advanced our understanding of metabolic reprogramming associated with ICD and provided valuable insights for clinical translation, especially for relapsed or resistant cancer patients [125]. Multi-omics analyses help identify unique metabolic vulnerabilities and biomarkers that can guide precise therapeutic interventions, improving treatment outcomes [126]. Currently, metabolomics is routinely employed to identify metabolic signatures predictive of therapeutic responses, enabling clinicians to optimize ICD-inducing therapies tailored to individual metabolic profiles.

### 6.6. Clinical Applications and Success Stories of Combining ICD and Metabolic Inhibitors

Several clinical studies have demonstrated the effectiveness of combining ICD inducers with metabolic inhibitors, achieving notable therapeutic successes. For example, drugs such as metformin (a glycolysis inhibitor), epacadostat (IDO1 inhibitor), and etomoxir (fatty acid oxidation inhibitor) have shown promising results when combined with ICD-based immunotherapies [127,128,129]. Clinical trials utilizing metformin combined with immune checkpoint inhibitors have achieved improved patient survival rates and tumor regression in melanoma and lung cancer [130]. Similarly, IDO1 inhibitors such as epacadostat, in combination with anti-PD-1 antibodies, have demonstrated encouraging efficacy and safety profiles in advanced melanoma clinical trials [131]. These clinical examples highlight the translational potential and feasibility of combining metabolic modulation and ICD-based immunotherapy strategies.

### 6.7. Genetic Strategies to Enhance ICD: CRISPR/Cas9

The emergence of advanced genetic engineering tools, particularly CRISPR/Cas9 technology, offers innovative avenues for enhancing ICD and metabolic reprogramming. CRISPR/Cas9-based gene editing enables precise modifications of genes involved in tumor metabolism and immune regulation, allowing for enhanced ICD induction and improved immune responses [132]. For instance, CRISPR-mediated knockout of glycolytic enzymes such as hexokinase 2 (HK2) or lactate dehydrogenase A (LDHA) has been demonstrated to sensitize tumors to immunotherapy by alleviating immunosuppressive lactate accumulation [133]. In preclinical models, targeted gene editing has successfully improved tumor immunogenicity and synergized with ICD-inducing treatments, indicating promising translational potential for clinical applications.

## 7. Conclusions and Future Perspectives

The integration of metabolic reprogramming with ICD presents a promising frontier in cancer immunotherapy. Tumor cells exhibit significant metabolic alterations that support their rapid growth and survival, while simultaneously reshaping the TME and influencing immune responses. The ability to exploit these metabolic changes—such as disruptions in serine metabolism, glycolysis, lipid metabolism, and mitochondrial dysfunction—offers a new approach to enhancing ICD-induced immune activation. By targeting these metabolic pathways, we can increase the immunogenicity of tumor cell death, promote immune responses, and improve the effectiveness of cancer therapies.

The critical role of serine metabolism, for example, in modulating T-cell proliferation and supporting immune responses highlights the importance of metabolic regulators in controlling immune function. Similarly, mitochondrial dysfunction and the resulting release of DAMPs play a central role in activating immune cells and promoting tumor-specific immunity. Combining metabolic inhibitors with ICD inducers, such as ferroptosis inducers or glycolysis inhibitors, could significantly enhance tumor cell death while simultaneously boosting immune responses. Additionally, personalized approaches based on multi-omics data, including genomics, proteomics, metabolomics, and transcriptomics, offer the potential for optimizing ICD-based therapies tailored to the specific metabolic characteristics of each patient’s tumor. This could provide more targeted and effective treatment strategies, improving patient outcomes and overcoming the challenge of tumor heterogeneity.

The future of cancer immunotherapy lies in the comprehensive understanding of how metabolism intersects with immune activation and ICD. Multi-omics technologies will continue to play a vital role in elucidating the complex interactions within the TME and immune system. Furthermore, the integration of personalized treatment strategies, including metabolic profiling, ICD-based therapies, and molecular imaging, will allow for real-time monitoring and dynamic adjustments to therapy, ensuring long-term efficacy. As research progresses, the potential to combine ICD with metabolic modulators, immune checkpoint inhibitors, and novel vaccine strategies offers a transformative approach to cancer treatment, making it possible to develop more precise, durable, and effective therapies that improve the prognosis for cancer patients.

In conclusion, the synergy between ICD and metabolic reprogramming holds immense promise for revolutionizing cancer immunotherapy. By leveraging metabolic and immunological insights, we can develop innovative, personalized treatment approaches that overcome immune evasion, enhance the antitumor immune response, and ultimately improve cancer treatment outcomes. The future of cancer immunotherapy lies in the integration of these approaches to provide patients with more effective, tailored therapies that address the complexities of cancer metabolism and immune function.

## Data Availability

No new data were created or analyzed in this study.

## Abbreviations

The following abbreviations are used in this manuscript:

ICD	Immunogenic cell death
DAMPs	Damage-associated molecular patterns
HMGB1	High-mobility group box 1
CRT	Calreticulin
TME	Tumor microenvironment
DCs	Dendritic cells
CTLs	Cytotoxic T lymphocytes
TAAs	Tumor-associated antigens
ROS	Reactive oxygen species
ER	Endoplasmic reticulum
ATP	Adenosine triphosphate
P2X7	P2X Purinoceptor 7
NLRP3	NLR family pyrin domain-containing protein 3
IL-1β	Interleukin-1β
TLR4	Toll-like receptor 4
RAGE	Receptor of advanced glycation endproduct
OXPHOS	Oxidative phosphorylation
mtDNA	Mitochondrial DNA
cGAS	cyclic GMP-AMP synthase
STING	Stimulator of interferon genes
PRRs	Pattern recognition receptors
FASN	Fatty acid synthase
ACSL4	acyl-CoA synthetase long-chain family member 4
GPX4	Glutathione peroxidase 4
PDT	Photodynamic therapy
4-HNE	4-hydroxy-2-nonenal
UPR	Unfolded protein response
DON	6-diazo-5-oxo-L-norleucine
IDO1	2,3-dioxygenase 1
SAMe	S-adenosylmethionine
PD-1	Programmed death-1
PD-L1	Programmed cell death ligand 1
AMPK	Adenosine 5′-monophosphate-activated protein kinase
CRISPR/Cas9	Clustered regularly interspaced short palindromic repeats—CRISPR-associated protein 9
RIPK1	Receptor-interacting protein kinase 1
RIPK3	Receptor-interacting protein kinase 3
MLKL	Mixed lineage kinase domain-like protein
LDHA	Lactate dehydrogenase A
HK2	Hexokinase 2
